# Congenital sideroblastic anemia model due to *ALAS2* mutation is susceptible to ferroptosis

**DOI:** 10.1038/s41598-022-12940-9

**Published:** 2022-05-30

**Authors:** Koya Ono, Tohru Fujiwara, Kei Saito, Hironari Nishizawa, Noriyuki Takahashi, Chie Suzuki, Tetsuro Ochi, Hiroki Kato, Yusho Ishii, Koichi Onodera, Satoshi Ichikawa, Noriko Fukuhara, Yasushi Onishi, Hisayuki Yokoyama, Rie Yamada, Yukio Nakamura, Kazuhiko Igarashi, Hideo Harigae

**Affiliations:** 1 Department of Hematology, Tohoku University Graduate School of Medicine, Sendai, Japan; 2 Laboratory Diagnostics, Tohoku University Hospital, Sendai, Japan; 3 Department of Biochemistry, Tohoku University Graduate School of Medicine, Sendai, Japan; 4 Department of Rheumatology, Tohoku University Graduate School of Medicine, Sendai, Japan; 5 Tohoku Electronic Industrial Co., Ltd., Sendai, Japan; 6 Cell Engineering Division, RIKEN BioResource Research Center, Tsukuba, Ibaraki Japan

**Keywords:** Molecular biology, Diseases, Pathogenesis

## Abstract

X-linked sideroblastic anemia (XLSA), the most common form of congenital sideroblastic anemia, is caused by a germline mutation in the erythroid-specific 5-aminolevulinate synthase (*ALAS2*) gene. In XLSA, defective heme biosynthesis leads to ring sideroblast formation because of excess mitochondrial iron accumulation. In this study, we introduced *ALAS2* missense mutations on human umbilical cord blood-derived erythroblasts; hereafter, we refer to them as XLSA clones. XLSA clones that differentiated into mature erythroblasts showed an increased frequency of ring sideroblast formation with impaired hemoglobin biosynthesis. The expression profiling revealed significant enrichment of genes involved in ferroptosis, which is a form of regulated cell death induced by iron accumulation and lipid peroxidation. Notably, treatment with erastin, a ferroptosis inducer, caused a higher proportion of cell death in XLSA clones. XLSA clones exhibited significantly higher levels of intracellular lipid peroxides and enhanced expression of BACH1, a regulator of iron metabolism and potential accelerator of ferroptosis. In XLSA clones, BACH1 repressed genes involved in iron metabolism and glutathione synthesis. Collectively, defective heme biosynthesis in XLSA clones could confer enhanced BACH1 expression, leading to increased susceptibility to ferroptosis. The results of our study provide important information for the development of novel therapeutic targets for XLSA.

## Introduction

Sideroblastic anemia is composed of a group of congenital and acquired disorders that share the characteristic presence of bone marrow ring sideroblasts, which represent excess mitochondrial deposition of iron^1,2^. In adults, these syndromes are often found to be related to myelodysplastic syndrome (MDS), which results from a mutation in the RNA-splicing machinery component splicing factor 3b, subunit 1 (*SF3B1*) gene^3^. On the other hand, the congenital forms of sideroblastic anemia (congenital sideroblastic anemia: CSA) are rare and constitute a diverse class of inherited disorders^1,2^. CSA has been considered to be caused by a mutation in the genes involved in heme biosynthesis, iron-sulfur cluster biosynthesis, and mitochondrial protein synthesis^2,4^.

X-linked sideroblastic anemia (XLSA) is the most common form of CSA and is caused by germline mutations in the erythroid-specific 5-aminolevulinate synthase (*ALAS2*) gene, which encodes the first and rate-limiting enzyme of heme biosynthesis^1,4^. The enzyme converts glycine and succinyl-coenzyme A to 5-aminolevulinic acid (ALA) and requires pyridoxal 5′-phosphate (vitamin B6) as a cofactor^1,2,4^. Clinically, patients with XLSA are predominantly hemizygous males who present with the disease most commonly < 40 years and exhibit hypochromic microcytic anemia with a varying degree that is accompanied by systemic iron overload^1,2,4,5^. Heterozygous female carriers can also develop sideroblastic anemia caused by skewed X-chromosome inactivation, which is related to aging^6–12^. Most XLSA-associated mutations are missense substitutions that result in a loss of protein functionality, whereas mutations in the *ALAS2* regulatory region have also been reported^2,4,13,14^. As different *ALAS2* missense mutations show different effects on the protein’s function, XLSA patients demonstrate varying degrees of disease severity^15^. Even female patients with macrocytic sideroblastic anemia due to heterozygous *ALAS2* mutations have been reported, which is attributed to the severe loss-of-function mutation in *ALAS2*^16,17^.

Although vitamin B6 is commonly used to treat XLSA, nearly half of the cases are unresponsive to treatment^4,5,18^. Additionally, oral ALA supplementation even failed to improve anemia in vitamin B6-refractory XLSA^19^. Thus, research of novel therapeutic strategies by clarifying the molecular basis of ring sideroblasts is required. So far, several attempts to establish the disease model of XLSA have been reported. In *Alas2*-knockout and -knockdown mice^20–22^, erythropoiesis did not proceed to the stage of ring sideroblast formation, resulting in death due to severe anemia. Similarly, *Alas2*-knockout embryonic stem cells^23^ did not yield ring sideroblasts. We recently reported the establishment of human ring sideroblasts based on induced pluripotent stem (iPS) cells derived from an XLSA patient^24^ as well as CRISPR/Cas9-based targeted disruption of the *ALAS2* regulatory region using human iPS cell-derived proerythroblast cell line (HiDEP-1)^22^. Through analysis of the latter XLSA model, we found that ring sideroblasts caused accumulation of anti-apoptotic enzymes such as heat shock protein 70 (HSP70), which indicated that the formation of ring sideroblasts might be a consequence of a cytoprotective reaction^22^. However, the detailed molecular mechanism by which excess mitochondrial iron accumulation confers impaired erythrocyte production that leads to the onset of anemia has remained elusive. Another issue was that iron supplementation of the media was necessary for ring sideroblast formation, and iron toxicity could affect the phenotype of the models. Additionally, our previous XLSA models expressed fetal hemoglobin^22,24^; thus, further characterization of the new XLSA model based on erythroblasts expressing adult hemoglobin was desired.

In this study, we aimed to create a new XLSA model based on human umbilical cord blood-derived erythroid progenitors (HUDEP-2) that synthesizes adult hemoglobin^25^. Thereafter, we investigated the molecular mechanism underlying the premature erythroid death in XLSA and explored novel therapeutic approach.

## Results

### ALAS2 R170L and R170H mutations result in impaired hemoglobin biosynthesis

To establish novel in vitro XLSA models, we employed the homology-directed CRISPR/Cas9 technology using HUDEP-2 cells, which showed a male (XY) karyotype and synthesized adult hemoglobin^25^. We aimed to introduce the single-nucleotide mutations in *ALAS2* that substitute arginine at amino acid residue 170, one of the XLSA hot spots, into leucine (R170L) or histidine (R170H) (Supplementary Fig. S1a and b). These mutations reportedly show different effects on the protein’s function: patients with *ALAS2* R170H are more refractory to vitamin B6 treatment^5,18,26^.

**Figure 1:**
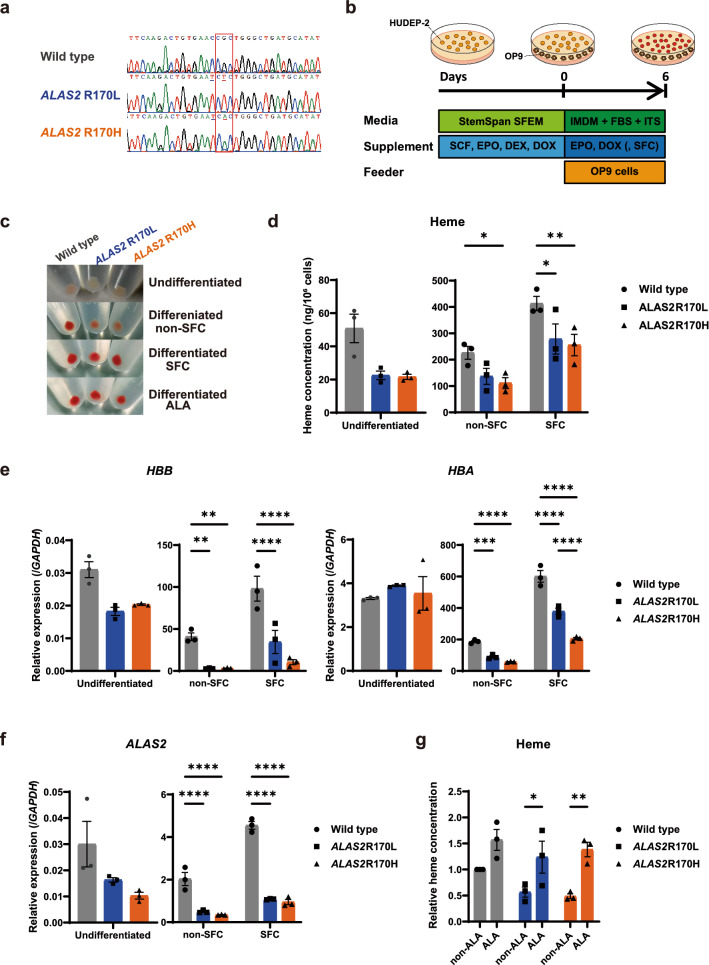
Establishment of HUDEP-2 cells harboring *ALAS2* R170L and R170H mutations. (**a**) Sanger sequencing around amino acid residue 170 of *ALAS2* (*ALAS2* R170, indicated by a red rectangle) in HUDEP-2 clones. Underlined nucleotides are substituted after CRISPR/Cas9. (**b**) Protocol for erythroid differentiation of HUDEP-2. DEX, dexamethasone; DOX, doxycycline; EPO, erythropoietin; FBS, fetal bovine serum; IMDM, Iscove’s Modified Dulbecco’s Medium; ITS, insulin-transferrin-selenium; SCF, stem cell factor; SFC, sodium ferrous citrate; SFEM, serum-free expansion medium. (**c**) Cell pellets of undifferentiated and differentiated HUDEP-2 clones. The effect of supplementation of SFC or 5-aminolevulinic acid (ALA) has also been demonstrated. Representative images of three independent experiments are presented. (**d**) Intracellular heme concentration of HUDEP-2 clones (n = 3). (**e**, **f**) Quantitative reverse transcriptase-polymerase chain reaction analysis for *HBB*, *HBA* (**e**) and *ALAS2* (**f**), expression relative to *GAPDH* in HUDEP^WT^, HUDEP^R170L^, and HUDEP^R170H^ (n = 3). (**g**) Intracellular heme concentration of HUDEP^WT^, HUDEP^R170L^, and HUDEP^R170H^ after 6 day differentiation with or without ALA supplementation (n = 3). The values are normalized relative to the mean value of undifferentiated HUDEP^WT^. In (**d**), (**e**), (**f**), and (**g**), the graphs were plotted using GraphPad Prism 9 (GraphPad Software, San Diego, CA, www.graphpad.com). The error bars represent the standard error of the mean. Each *P*-value was calculated using Tukey’s test (**d**, **e**, and **f**) or Šidák’s test (**g**) after a two-way analysis of variance. *P < 0.05, **P < 0.01, ***P < 0.001, ****P < 0.0001.

As shown in Fig. 1a, we successfully established “XLSA clones” harboring the *ALAS2* R170L and R170H mutations (also termed as HUDEP^R170L^ and HUDEP^R170H^, respectively), and off-target mutations were not identified (potential off-target sites are listed in Supplementary Table S1). Next, we induced wild-type HUDEP-2 cells (termed as HUDEP^WT^) and XLSA clones (HUDEP^R170L^ and HUDEP^R170H^) to undergo erythroid differentiation by co-culturing with OP9 cells for 6 days (Fig. 1b)^22^. We optionally supplied sodium ferrous citrate (SFC) in the differentiation medium based on our previous findings that SFC promoted erythroid differentiation of HiDEP-1 cells as well as cord blood CD34^+^ cell-derived primary erythroblasts^22^. We confirmed that the pellets of cells which differentiated in the SFC-supplemented medium turned more reddish than those in the non-SFC-supplemented medium (Fig. 1c). Meanwhile, the cell pellet colors of XLSA clones were less reddish than those of HUDEP^WT^ after erythroid differentiation (Fig. 1c). Concomitantly, intracellular heme concentration and the expressions for globin genes (*HBB* and *HBA*) were increased after differentiation in HUDEP^WT^ and XLSA clones, whereas they were significantly suppressed in XLSA clones compared with those in HUDEP^WT^ (Fig. 1d and e). Intriguingly, we also noticed significant downregulation of *ALAS2* expression in both erythroid differentiation-uninduced and -induced XLSA clones (Fig. 1f). We treated the differentiated cells with the transcription inhibitor actinomycin D and found that the treatment significantly enhanced *ALAS2* messenger RNA (mRNA) decay in XLSA clones compared with that in HUDEP^WT^ (Supplementary Fig. S2). Our findings indicate that *ALAS2* R170 mutations have no effect on its transcription but rather destabilize *ALAS2* mRNA, resulting in its posttranscriptional degradation. Regarding ALA supplementation of the medium, we found that ALA promoted hemoglobin biosynthesis and enhanced *HBB* expression during differentiation of XLSA clones, although the latter was not statistically significant (Fig. 1c,g, Supplementary Fig. S3). Expression levels of *ALAS2* in HUDEP^WT^ were suppressed after ALA supplementation, but those in XLSA clones were unchanged (Supplementary Fig. S3). Although heme concentration reportedly does not affect *ALAS2* expression^27^, the results suggest a heme-independent feedback mechanism of *ALAS2*, which could be impaired by *ALAS2* R170 mutations. Collectively, our findings confirmed that, in XLSA clones, heme biosynthesis is compromised due to ALA deficiency.

### *ALAS2* R170L and R170H mutations result in impaired erythroid differentiation and ring sideroblast formation

**Figure 2:**
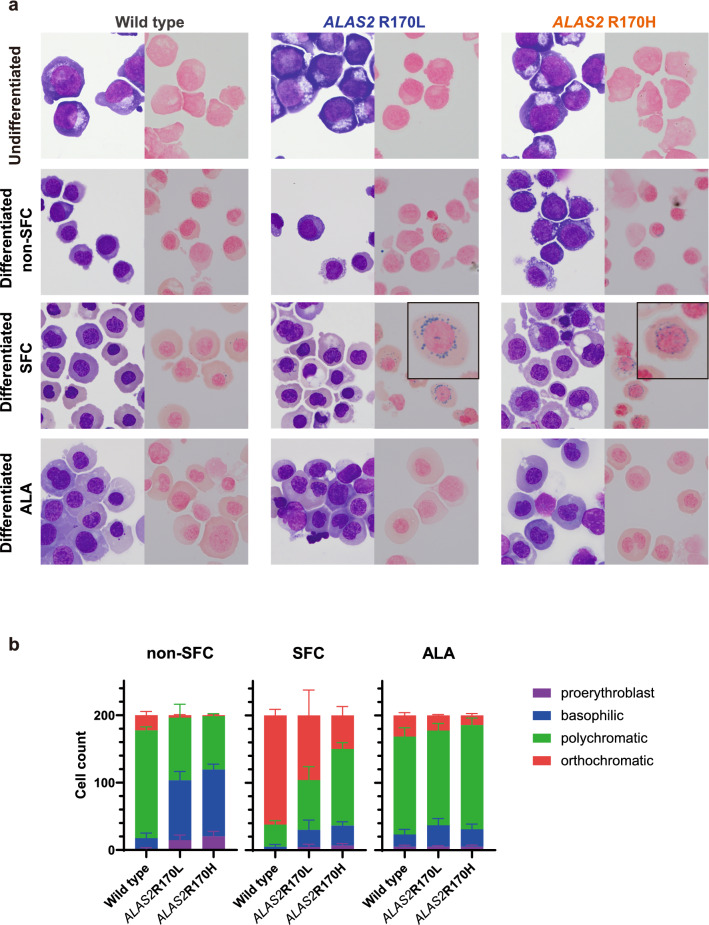
*ALAS2* mutations result in impaired erythroid differentiation and ring sideroblast formation. (**a**) May-Giemsa (each of left panel) and Prussian blue staining (each of right panel) of HUDEP^WT^, HUDEP^R170L^, and HUDEP^R170H^. Ring sideroblasts are zoomed in the subpanels. The representative images of three independent experiments are shown. (**b**) Count of erythroid progenitor cells derived from HUDEP-2 clones after 6 day differentiation (n = 3). May-Giemsa staining was used for quantification. The graphs were plotted using GraphPad Prism 9 (GraphPad Software, San Diego, CA, www.graphpad.com). ALA, 5-aminolevulinic acid; SFC, sodium ferrous citrate.

**Figure 3:**
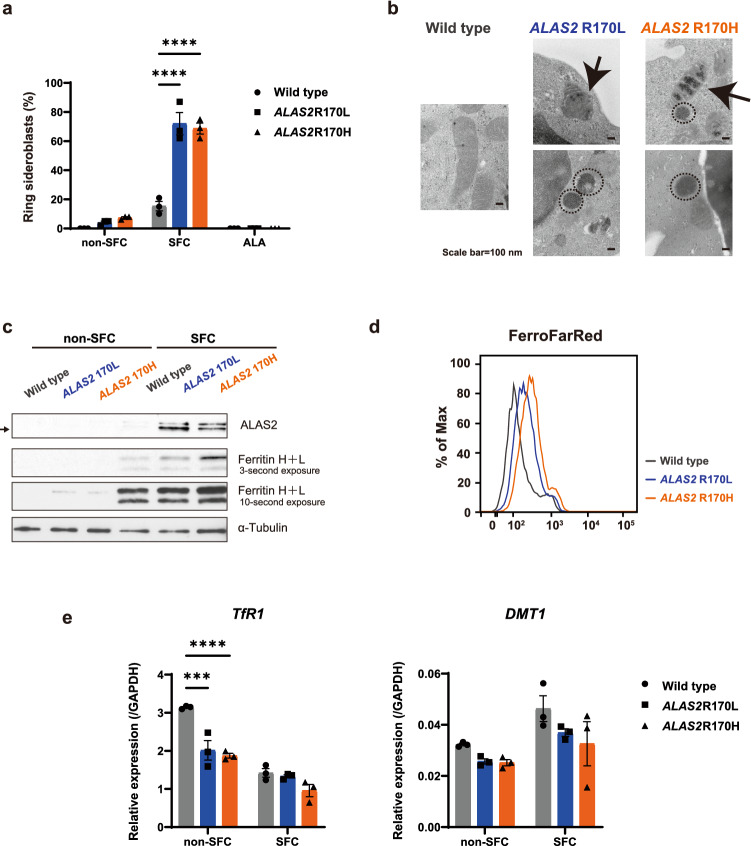
*ALAS2* mutations result in iron overload. (**a**) Count of ring sideroblasts of HUDEP-2 clones after 6 day differentiation without sodium ferrous citrate (SFC), with SFC and with 5-aminolevulinic acid (ALA) (n = 3). Error bars represent the standard error of the mean. Each *P* value was calculated using Tukey’s test after a two-way analysis of variance. ****P < 0.0001. (**b**) Images of electron microscopy in HUDEP-2 clones after differentiation with SFC. Arrows indicate aberrant mitochondrial iron deposits. The portions surrounded by a dashed line indicate mitochondrial spheroids. (**c**) Western blotting for ALAS2, ferritin and α-tubulin in HUDEP-2 clones after differentiation with or without SFC. The cropped gel images are delineated, and the uncropped images can be found in Supplementary Fig. S7. In (**b**) and (**c**), the representative images of three independent experiments are shown. (**d**) Intracellular labile iron of HUDEP-2 clones after differentiation with SFC. The fluoroprobe FerroFarRed was used for a marker of intracellular labile iron. The representative histogram of three independent experiments, plotted using FlowJo version 7.6.5 software (TreeStar, Ashland, OR, www. flowjo. com), is shown. (**e**) Quantitative reverse transcriptase-polymerase chain reaction analysis for *TfR1* and *DMT1*, expression relative to *GAPDH* in HUDEP^WT^, HUDEP^R170L^, and HUDEP^R170H^ (n = 3). In a and e, graphs were plotted using GraphPad Prism 9 (GraphPad Software, San Diego, CA, www.graphpad.com).

We explored whether *ALAS2* mutations affect the erythroid proliferation and differentiation in XLSA clones. The cell numbers after erythroid differentiation were similar between XLSA clones and HUDEP^WT^, suggesting that XLSA clones proliferated normally during erythroid differentiation (Supplementary Fig. S4). On the other hand, XLSA clones showed a reduced expression of CD71 and CD49d compared to those of HUDEP^WT^ (Supplementary Fig. S5). This finding seemingly indicates that XLSA clones proceed to a more mature erythroid stage compared to HUDEP^WT^; however, it could have been influenced by an iron-overloaded condition. We alternatively tracked the differentiation by cell morphology and found that the proportion of mature erythroblasts is decreased in XLSA clones (Fig. 2a,b). Heme is an important regulator of erythropoiesis^20^, and its deficiency may have impaired the differentiation of XLSA clones. Notably, almost 70% of HUDEP^R170L^ and HUDEP^R170H^ exhibited the phenotype of typical ring sideroblasts after erythroid differentiation with SFC (Figs. 2a, 3a). ALA supplementation promoted proper differentiation of XLSA clones (Fig. 2a,b), and ring sideroblasts reverted to normal erythroblasts after the addition of ALA (Fig. 3a). Electron microscopy revealed that XLSA clones after erythroid differentiation with SFC showed aberrant mitochondrial iron deposits and a remarkable increase in the mitochondrial spheroids, indicating a response to oxidative stress (Fig. 3b and Supplementary Fig. S6)^28^. In Western blotting, differentiated XLSA clones showed accumulation of ferritin and ALAS2 especially under conditions supplemented with SFC (Fig. 3c and Supplementary Fig. S7). We think that an iron-overloaded condition is responsible for their expression at the posttranscriptional level through the iron-responsive element/iron-responsive proteins (IRE/IRP) regulatory system^29^. Increased levels of intracellular labile iron were also noted in XLSA clones (Fig. 3d). We checked the expression levels of transferrin receptor 1 (*TfR1*) and divalent metal transporter 1 (*DMT1*) genes. Under iron-overloaded conditions, mRNAs of *TfR1* and *DMT1* are destabilized due to decreased IRP binding to IRE in the 3ʹ untranslated regions^29^. Unexpectedly, we found no significant change in the mRNA expression levels between XLSA clones and HUDEP^WT^ except for *TfR1* after differentiation without SFC (Fig. 3e). We postulate that multiple factors other than the IRE/IRP system could affect gene expression during erythroid differentiation and ring sideroblast formation^22^. Nevertheless, impaired erythroid differentiation and ring sideroblast formation represent the characteristics of erythroblasts of XLSA patients. Hence, the XLSA clones were considered suitable models of the disease.

### Increased susceptibility to ferroptosis in XLSA clones

**Figure 4:**
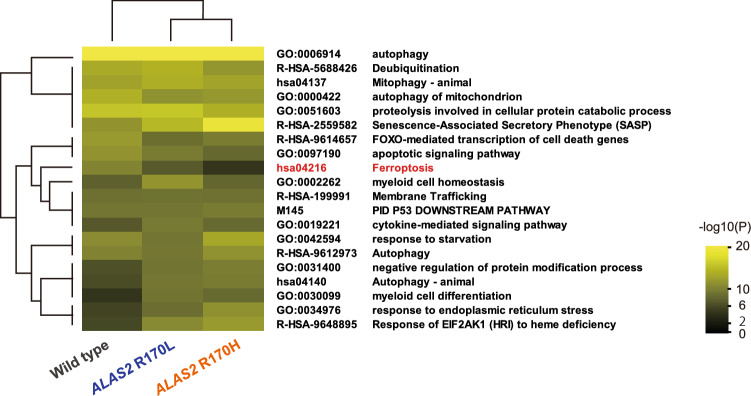
Genes associated with oxidative stress protection are differentially expressed during erythroid differentiation. Enrichment analysis of HUDEP-2 clones using Metascape^59^. After 6 day differentiation with SFC, 1265, 1750, and 1386 genes were upregulated (> 2-fold) for HUDEP^WT^, HUDEP^R170L^, and HUDEP^R170H^, respectively, compared with the undifferentiated HUDEP^WT^. These genes were analyzed, and enriched gene clusters were displayed as a heatmap that shows hypergeometric *P*-values of each annotated term. The heatmap is visualized using Java TreeView version 1.1.6r4 (jtreeview.sourceforge.net). EIF2AK1, eukaryotic translation initiation factor 2-alpha kinase 1; HRI, heme-regulated inhibitor.

To reveal the molecular consequences of ring sideroblast formation, we compared the gene expression profile of HUDEP^WT^ and XLSA clones after 6 days of erythroid differentiation with that of undifferentiated HUDEP^WT^. We found 1265, 1750, and 1386 genes that were upregulated (> 2-fold) for HUDEP^WT^, HUDEP^R170L^, and HUDEP^R170H^, respectively (Fig. 4). Gene ontology analysis of the upregulated gene ensemble showed a significant (*p* < 0.01) enrichment of genes associated with autophagy, endoplasmic reticulum stress responses, eukaryotic translation initiation factor 2 alpha kinase 1 signaling pathway, forkhead box O-mediated cell death, p53 pathway, and ferroptosis. These pathways are commonly associated with the cellular stress response, especially protection from oxidative stress. We speculated that these genes contributed to the suppression of cellular damage during heme biosynthesis in the erythroblasts. In this study, we focused on the genes associated with “ferroptosis,” which was a newly identified form of regulated cell death that is dependent on iron accumulation and lipid peroxidation^30^. We hypothesized that XLSA clones are prone to ferroptosis via the restriction of the expression of ferroptosis-inhibitory genes.

**Figure 5:**
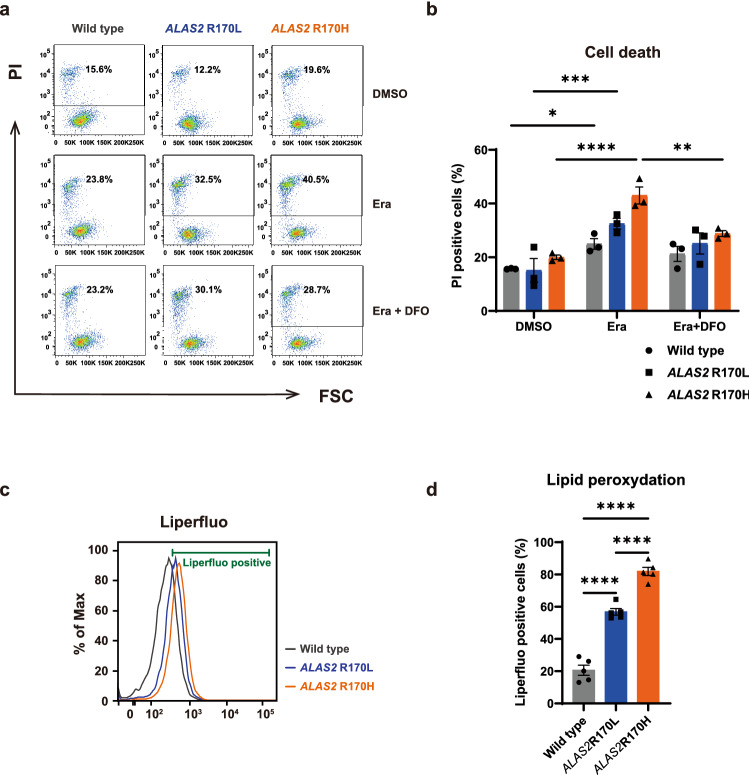
XLSA clones show higher ferroptosis susceptibility. (**a**, **b**) Cell death assessment of HUDEP^WT^, HUDEP^R170L^, and HUDEP^R170H^ after 6 day differentiation with sodium ferrous citrate (SFC) followed by 24 h treatment with 100 μM erastin (Era; Selleck Chemicals, Houston, TX) with or without 100 μM deferoxamine (DFO; Sigma-Aldrich, St. Louis, MO). Using flow cytometry, propidium iodide (PI)-positive cells were defined as dead cells. The representative scattergram (**a**) and quantification of dead cells (**b**, n = 3) are shown. DMSO, dimethyl sulfoxide; FSC, forward scatter. (**c**, **d**) Lipid peroxidation of HUDEP-2 clones after differentiation with SFC. The fluoroprobe Liperfluo was used for a marker of lipid peroxidation. The representative histogram (**c**) and quantification of Liperfluo positive cells (**d**, n = 5) are shown. In (**a**) and (**c**), the data were analyzed using FlowJo version 7.6.5 software (TreeStar, Ashland, OR, www. flowjo. com). In (**b**) and (**d**), the graphs were plotted using GraphPad Prism 9 (GraphPad Software, San Diego, CA, www.graphpad.com). The error bars represent the standard error of the mean. Each *P* value was calculated using Dunnett’s test after a two-way analysis of variance (**b**) or Tukey’s test after a one-way analysis of variance (**d**). *P < 0.05, **P < 0.01, ***P < 0.001, ****P < 0.0001.

To validate the hypothesis, we treated XLSA clones with erastin, which induces ferroptosis^30^. As shown in Fig. 5a, the XLSA clones showed a higher proportion of cell death after erastin treatment, suggesting an increase in ferroptosis susceptibility. Furthermore, cell viability was improved after treatment with deferoxamine (DFO), an iron chelator (Fig. 5b). The concentration of intracellular lipid peroxides that eventually execute ferroptosis was significantly higher in XLSA clones (Fig. 5c,d; and Supplementary Fig. S8). The level of intracellular lipid peroxides was significantly higher in HUDEP^R170H^ than in HUDEP^R170L^, which reflected the clinical refractoriness of XLSA patients harboring *ALAS2* R170H^5,18,26^. These findings suggest that the XLSA clones were accompanied by increased lipid peroxidation and higher susceptibility to ferroptosis.

### BACH1 contributes to increased susceptibility of ferroptosis in XLSA

**Figure 6:**
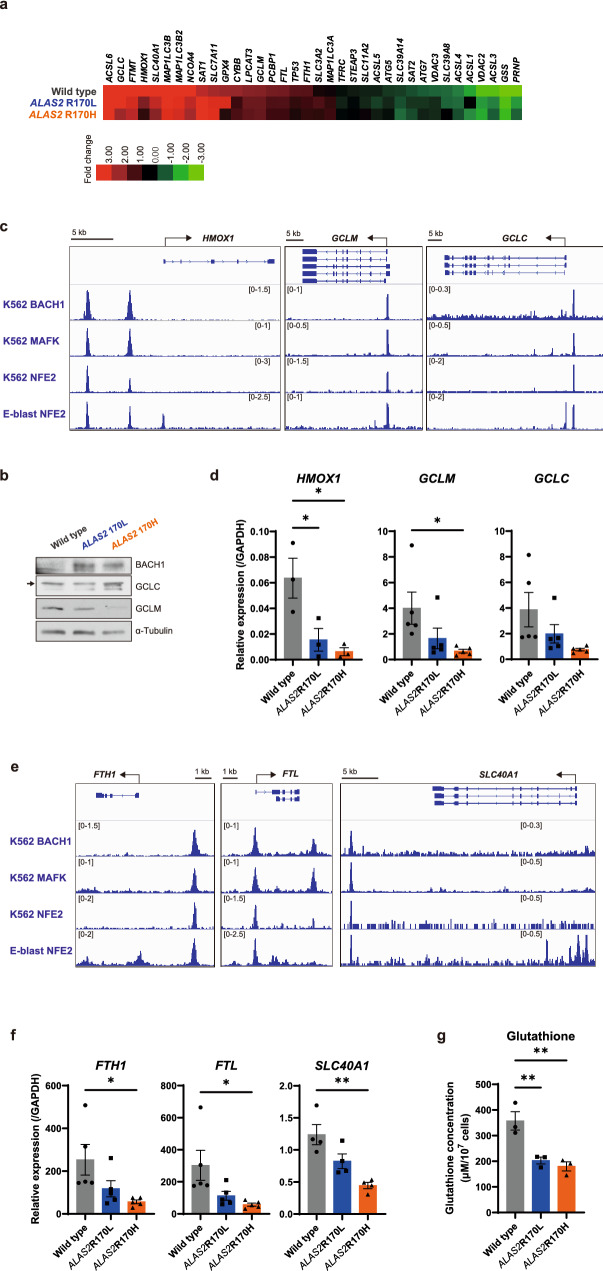
XLSA clones show transcriptional alterations of the genes associated with glutathione synthesis. (**a**) Expression profiles of the genes registered to hsa04216 (ferroptosis pathway) of Kyoto Encyclopedia of Genes and Genomes in HUDEP^WT^, HUDEP^R170L^, and HUDEP^R170H^ after 6 day differentiation with sodium ferrous citrate (SFC). The genes are arranged from the bottom in the order of the fold change of differentiated HUDEP^WT^ to undifferentiated HUDEP^WT^. The heatmap is visualized using Java TreeView version 1.1.6r4 (jtreeview.sourceforge.net). (**b**) Western blotting for BACH1, GCLC, GCLM, and α-tubulin in HUDEP-2 clones after differentiation with SFC. Representative images of three independent experiments are presented. The cropped gel images are delineated, and the uncropped images can be found in Supplementary Fig. S9. (**c**, **e**) Chromatin immunoprecipitation with sequencing (ChIP-seq) analysis of the binding of BACH1 and MAFK for the gene region in K562 for *HMOX1*, *GCLM*, and *GCLC* (**c**); and *FTH1*, *FTL*, and *SLC40A1* (**e**). We used ChIP-seq data from GEO (Gene Expression Omnibus) data set: BACH1 in K562, GSM935576; MAFK in K562, GSM935311; NFE2 in K562, GSM935414; and NFE2 in human erythroblasts, GSM1427076. E-blast, erythroblast. (**d**, **f**) Quantitative reverse transcriptase-polymerase chain reaction analysis for *HMOX1*, *GCLM*, and *GCLC* (**d**); and *FTH1*, *FTL*, and *SLC40A1* (**f**) in HUDEP-2 clones after differentiation with SFC. The data were normalized relative to the *GAPDH* expression levels. (**g**) Intracellular glutathione concentration of HUDEP-2 clones after differentiation with SFC (n = 3). In (**d**), (**f**), and (**g**), the graphs were plotted using GraphPad Prism 9 (GraphPad Software, San Diego, CA, www.graphpad.com). The error bars represent the standard error of the mean. Each *P* value was calculated using Tukey’s test after a one-way analysis of variance. *P < 0.05, **P < 0.01, ***P < 0.001, ****P < 0.0001.

Next, we explored the basis for increased ferroptosis susceptibility in the XLSA clones. We noticed a downregulation of a subset of ferroptosis-inhibitory genes as well as iron/heme regulatory genes in XLSA clones compared with that in HUDEP^WT^ (Fig. 6a). Based on recent reports, ferroptosis could be controlled by BACH1 (BTB and CNC homology 1), which is known as a heme-regulated transcriptional repressor^31^. Thus, we conducted Western blotting that confirmed the abundant BACH1 protein expression in the XLSA clones (Fig. 6b and Supplementary Fig. S9). Referring to the previous chromatin immunoprecipitation with sequencing (ChIP-seq) data in K562 cells^32^ (Gene Expression Omnibus (GEO) data set: BACH1, GSM935576; and MAFK, GSM935311), we detected peaks of BACH1 and its partner MAFK in the regulatory sites of ferroptosis-related genes, such as heme oxygenase 1 (*HMOX1*), glutamate-cysteine ligase modifier subunit (*GCLM*)*,* and glutamate-cysteine ligase catalytic subunit (*GCLC*) (Fig. 6c), as well as key regulators of iron metabolism, such as ferritin heavy chain 1 (*FTH1*), ferritin light chain (*FTL*), and solute carrier family 40 member 1 (*SLC40A1*) (Fig. 6e). The transcriptional regulation of K562 is representative of human erythroblasts because the erythroid-specific transcription factor, nuclear factor, erythroid 2 (NFE2) binds to the similar DNA sites in K562 and human erythroblasts (GEO data set: NFE2 in K562, GSM935414; and NFE2 in human erythroblasts, GSM1427076) (Fig. 6c,e). We also confirmed that NFE2, which competes with BACH1 to activate genes, binds to similar sites as BACH1 and MAFK (Fig. 6c,e).

Quantitative reverse transcriptase-polymerase chain reaction (RT-PCR) analyses confirmed the reduced expressions of these genes in XLSA clones, suggesting that BACH1 binds to the regulatory sites of the target genes and negatively regulates their expression (Fig. 6d,f). We ruled out the notion that the XLSA clones’ less-differentiated state contributed to the lower expression of these genes: The expression level of *HMOX1* were much lower in XLSA clones after 6 day differentiation than in HUDEP^WT^ after 4.5 day differentiation, which exhibits more immature morphology with the decreased proportion of mature erythroblasts (Supplementary Fig. S10a and b).

Notably, some of the genes downregulated by BACH1 were associated with glutathione synthesis (*GCLM* and *GCLC*). Glutathione is important for decreasing lipid peroxidation and suppressing ferroptosis^33^. *GCLM* and *GCLC* encode the two components of glutamate-cysteine ligase, the rate-limiting enzyme of glutathione synthesis^34^. As shown in Fig. 6g, the levels of intracellular glutathione concentrations were significantly decreased in the XLSA clones. Intriguingly, we found that the amounts of GCLM protein were significantly decreased in XLSA clones than those in HUDEP^WT^, whereas the GCLC protein levels were similar between the XLSA clones and HUDEP^WT^ (Fig. 6b). This implies that BACH1 could mediate the reduction of intracellular glutathione concentrations mainly by repressing *GCLM* expression.

## Discussion

In this study, we established novel in vitro XLSA models that have potential advantages over previous models based on HiDEP-1 cells; the newly established XLSA clones synthesized adult hemoglobin^25^. Additionally, a majority (approximately 70%) of the cells formed ring sideroblasts after erythroid differentiation (Fig. 3a), whereas the frequency of ring sideroblast formation in the previous model was only 20%^22^. Because of a lack of specific markers, it would be difficult to sort the ring sideroblasts. Thus, the results derived from the new models may recapitulate the pathophysiology of human ring sideroblasts more precisely than those of previous models^22^.

To synthesize the XLSA clones, we introduced single-nucleotide mutations in *ALAS2* that substitute arginine at amino acid residue 170 into leucine (R170L) or histidine (R170H), which would result in a loss of protein functionality. We confirmed the decreased globin gene expression in the XLSA clones (Fig. 1e), which would be mediated by the enhanced BACH1 protein (Fig. 6b)^35^. We unexpectedly found that *ALAS2* expression is also downregulated in XLSA clones (Fig. 1f): results of actinomycin D treatment suggest that *ALAS2* R170 mutations destabilize *ALAS2* mRNA, causing its posttranscriptional degradation, although the precise mechanism remains unknown (Supplementary Fig. 2). Similar to the RNA-binding protein DiGeorge critical region-8 (DGCR8), which confers processing of pri-miRNA in a heme-dependent manner^36^, we speculate that there might be hitherto unrecognized mechanisms by which heme could affect mRNA stability for a subset of genes. We considered the possibility that BACH1 binds to the regulatory site of *ALAS2* and negatively controls its expression; however, no peaks of BACH1 and MAFK were detected around the *ALAS2* gene in reference to the Chip-seq data in K562 (Supplementary Fig. S11). We also ruled out the notion that delayed erythroid differentiation in XLSA clones is responsible for the low *ALAS2* expression: The expression levels of *ALAS2* in XLSA clones after 6 day differentiation are even lower than those in HUDEP^WT^ after 4.5 day differentiation, which exhibits more immature morphology (Supplementary Fig. S10a and b). Contrary to decreased mRNA levels, ALAS2 protein expression was significantly increased in XLSA clones (Fig. 3c), which contributed to a relatively sustained heme concentration: heme concentrations in XLSA clones were about 60%–70% of those in HUDEP^WT^ (Fig. 1d), whereas *ALAS2* mRNA levels in XLSA clones were about 20% of those in HUDEP^WT^ (Fig. 1f).

We also showed that expressions levels of *FTH1* and *FTL* in XLSA clones are suppressed via transcriptional regulation by BACH1 (Fig. 6f), whereas ferritin protein expression was increased in the XLSA clones (Fig. 3c). It has been known that IRE located at the 5′-untranslated region of *ALAS2*, *FTH1* and *FTL* is responsible for regulating their expression at the posttranscriptional level by the function of IRP^29^. Under iron-deficient conditions, the translation of *ALAS2, FTH1* and *FTL* is suppressed by the binding of IRP to the IRE. The IRP detach from the IRE under iron-overloaded conditions, resulting in their increased translation^29^, which may account for the increased levels of ALAS2 and ferritin proteins.

We tried to characterize XLSA clones and showed that the XLSA clones had increased lipid peroxidation and higher susceptibility to ferroptosis. Ferroptosis is triggered by oxidative stress via the Fenton reaction (the reaction of peroxides with Fe^2+^ to yield hydroxy radicals)-mediated lipid peroxidation; thus, it has been implicated various types of oxidative stress-related diseases such as myocardial diseases, renal failure, and neurodegenerative disorders^37–40^. Current evidence related to β-thalassemia and MDS, in which ineffective erythropoiesis is the hallmark of the diseases as well as in XLSA, has suggested a causal relationship between lipid peroxidation and iron-loading anemia^41–46^. As such, XLSA may have a potential link with ferroptosis. Considering that the cell viability was comparable between XLSA clones and HUDEP^WT^ after 6 days of differentiation (Fig. 5a,b), we speculate that the higher susceptibility of XLSA clones to ferroptosis impairs terminal erythroid maturation after enucleation. A recent study showed that hematopoietic cell-specific ablation of murine *Gpx4*, a representative anti-ferroptotic gene^47^, led to anemia, increased production of erythroid precursors in the bone marrow, and systemic iron overload that mimicked ineffective erythropoiesis^48^. This mouse model also exhibited lipid peroxidation and maturation defects in reticulocytes^48^, which might account for early death in *Gpx4*-deficient erythroid progenitors^49^. We believe that similar mechanisms operate in XLSA clones, where reduced glutathione synthesis causes lipid peroxidation. This hypothesis should be confirmed by refining the in vitro culture conditions that enable HUDEP-2 cells to differentiate into enucleated erythroid cells or by establishing an in vivo XLSA model.

**Figure 7:**
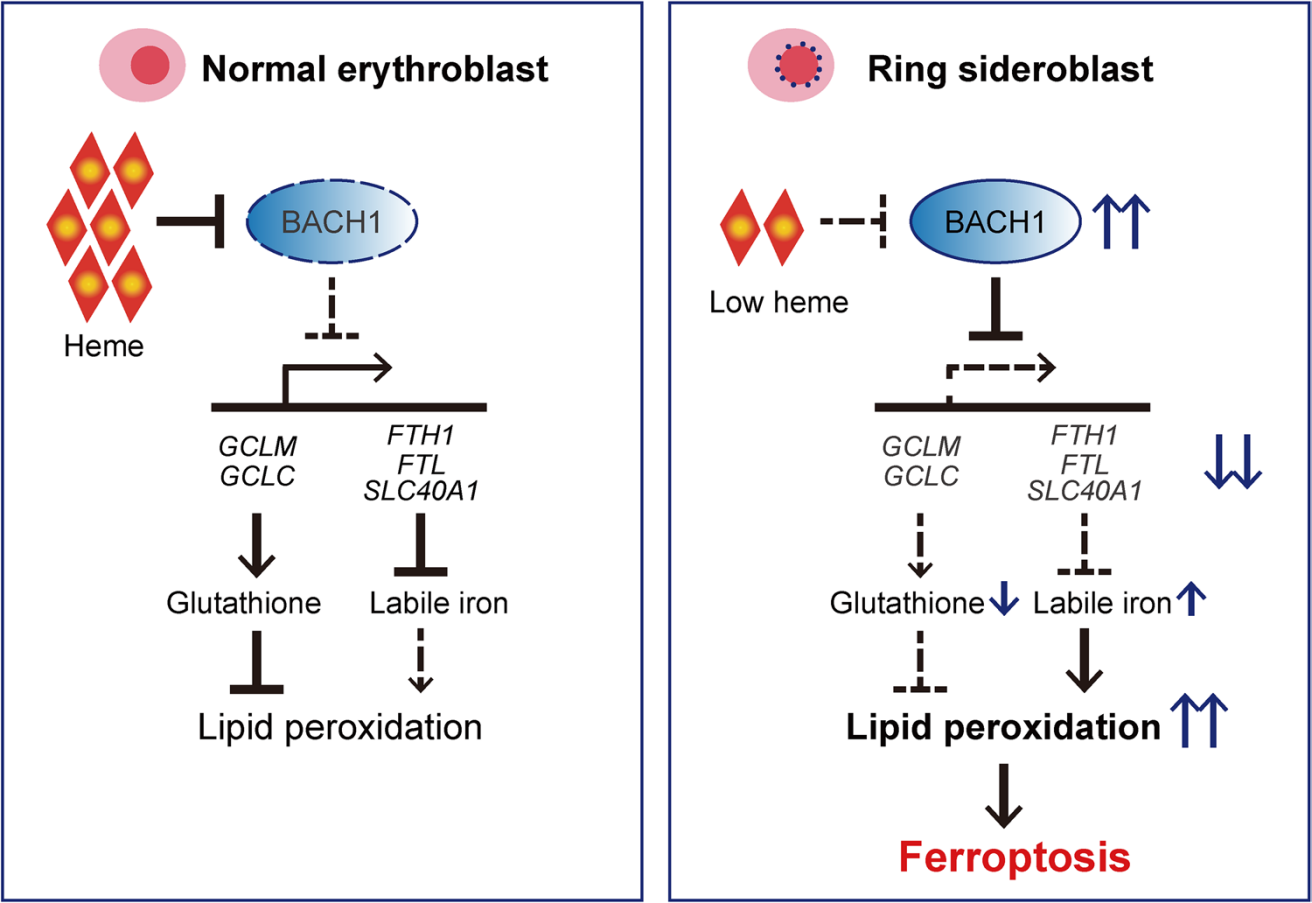
Schematic representation of the pathophysiology of XLSA. In XLSA, impaired heme biosynthesis enhances BACH1 expression, resulting in the alteration of the transcriptional program during differentiation, resulting in the suppression of genes associated with glutathione synthesis (i.e., *GCLM*) and iron metabolism (i.e., *FTH1* and *FTL*). Reduced glutathione synthesis causes higher levels of lipid peroxidation, leading to increased ferroptosis sensitivity.

We also showed that impaired heme biosynthesis in XLSA enhanced BACH1 levels, which altered the transcriptional program during differentiation. This resulted in the suppression of genes associated with glutathione synthesis and iron metabolism, leading to increased susceptibility to ferroptosis (Fig. 7). This is in line with previous observations based on murine embryonic fibroblasts (MEF)^31^. Western blotting analysis demonstrated that the GCLM protein level was significantly decreased in XLSA clones than in HUDEP^WT^, whereas GCLC protein levels were similar between XLSA clones and HUDEP^WT^ (Fig. 6b). This suggests that BACH1 could reduce intracellular glutathione levels mainly by repressing *GCLM* expression. A previous study has indicated that GCLC protein expression was unchanged even in BACH1-knockout MEF^31^, potentially accounting for the stable GCLC protein expression in certain circumstances.

One limitation is that we compared XLSA clones and HUDEP^WT^ at different stages of erythroid differentiation. We understand that it is best to sort erythroblasts at specific stages, but the cell surface markers may be affected by an iron-overloaded condition. We demonstrated that the expression levels of *ALAS2*, *HBB*, and *HMOX1* in XLSA clones after 6 day differentiation are even lower than those in HUDEP^WT^ after 4.5 day differentiation (Supplementary Fig. S10a and b). However, the results of the cell death assay, Western blotting, lipid peroxidation assay, or glutathione measurement are left to be verified using the differentiation-adjusted control. Thus, when the data are interpreted, we should consider possible artifacts derived from the different erythroid stage.

Another limitation is that our culture condition requires SFC to yield ring sideroblasts. Recently, an acquired sideroblastic anemia model was established via iPS cells derived from an *SF3B1*-mutant MDS patient^50^. In this model, up to 40% of cells developed ring sideroblasts after erythroid differentiation. Notably, their culture medium did not require SFC supplementation^50^; thus, the model seems physiological and suitable for analysis. Considering this, our culture conditions can be improved to characterize XLSA clones under SFC-free conditions.

Although we established two types of XLSA models (HUDEP^R170L^ and HUDEP^R170H^), the molecular basis of the clinical variability of XLSA remains unclear. Clinically, patients with *ALAS2* R170L are often responsive to vitamin B6 treatment, but those with *ALAS2* R170H are refractory to treatment^5,10,18,26^. Previous studies have yet to clarify the causative factors for this difference. Bacterially-expressed ALAS2 with R170L and R170H both show lower enzymatic activities than wild type ALAS2, which are improved by vitamin B6 treatment^5,10^. In the present study, the XLSA clone with *ALAS2* R170H, which represents the vitamin B6-refractory phenotype^5,18,26^, showed lower glutathione concentrations (Fig. 6g), increased levels of lipid peroxidation (Fig. 5d), and higher frequencies of cell death with erastin treatment (Fig. 5b). These suggest that the clinical severity of XLSA is linked to the susceptibility to ferroptosis of the erythroblasts. However, the intracellular heme concentration and BACH1 levels were comparable between HUDEP^R170L^ and HUDEP^R170H^ (Figs. 1d, 6b); thus, we considered the possibility that a pathway other than the heme-BACH1 axis increased ferroptosis susceptibility in HUDEP^R170H^. To address these questions, another in vitro model of XLSA should be established, such as R452H and R411C, which are other common *ALAS2* mutations^1,2,5^, or R163H and Y365H, which are associated with female patients with macrocytic sideroblastic anemia due to severe *ALAS2* loss-of-function mutation^16,17^.

Additionally, we should consider the impact of systemic iron metabolism in XLSA pathology. XLSA patients commonly exhibit systemic iron overload even in transfusion-free cases^1,51^. Accordingly, our model showed that the supplementation of SFC induced ring sideroblast formation in the XLSA clones. Altogether, there should be a mechanism through which excessive iron is transported into the patients’ erythroblasts in XLSA. We believe that other types of iron-loading anemia, such as β-thalassemia and MDS with ring sideroblasts, share similar pathogenic mechanisms with XLSA. In these conditions, a low hepcidin level plays a role in iron overload^52^. Hepcidin is a liver peptide that tightly regulates iron homeostasis by degrading ferroportin (encoded by *SLC40A1*) in the enterocytes and macrophages^52^. Recently, XLSA patients with low serum hepcidin levels and iron overload have been demonstrated^53^. In XLSA, insufficient hepcidin production and the subsequent upregulation of ferroportin may contribute to the increased iron uptake, resulting in ring sideroblast formation and subsequent ferroptosis. This issue needs to be investigated further using an appropriate in vivo model in the future.

Finally, a therapeutic strategy for XLSA that targets the ferroptosis pathway should be established. Considering that some pathological conditions associated with ferroptosis reportedly improve following treatment with ferroptosis inhibitors such as vitamin E^31,54,55^, we assume that the same approach could be useful for XLSA. Moreover, systemic iron overload can affect vitamin B6 responsiveness in XLSA patients; therefore, combination therapy with ferroptosis inhibitors and vitamin B6 could be a treatment option for refractory cases^56^.

In conclusion, we believe that this is the first study that has used the novel in vitro XLSA models that clearly demonstrated the involvement of ferroptosis in the pathophysiology of XLSA. Further studies are required to establish a therapeutic strategy targeted against ferroptosis and to clarify whether ferroptosis commonly contributes to the pathology of ineffective erythropoiesis.

## Methods

The study protocol was approved by the Committee for Safe Handling of Living Modified Organisms of Tohoku University Graduate School of Medicine. All methods were carried out in accordance with relevant guidelines and regulations.

### Cell culture

HUDEP-2 cells were derived from human umbilical cord blood CD34^+^ cells, which were obtained from the Stem Cell Resource Network in Japan (Banks at Miyagi, Tokyo, Kanagawa, Aichi, and Hyogo) through the RIKEN BioResource Research Center^25^. Informed consent for cord blood donation has been obtained. HUDEP-2 cells were established via viral transduction of transcription factor TAL1 and human papillomavirus-E6/E7^25^ and maintained in StemSpan serum-free expansion medium (STEMCELL Technologies, Vancouver, BC, Canada) supplemented with 50 ng/mL stem cell factor (PEPROTECH, Rocky Hill, NJ), 3 IU/mL erythropoietin (Kyowa Hakko Kirin, Tokyo, Japan), 1 μg/mL doxycycline (Sigma-Aldrich, St. Louis, MO), and 1 μM dexamethasone (Sigma-Aldrich). OP9 cells, obtained from the American Type Culture Collection (Manassas, VA), were maintained in an α minimum essential medium (Thermo Fisher Scientific, Waltham, MA) supplemented with 20% fetal bovine serum (FBS; Biological Industries USA, Cromwell, CT) and 1% penicillin–streptomycin (Sigma-Aldrich).

### Generation of cell lines harboring ALAS2 R170L and R170H mutations using CRISPR/Cas9

HUDEP-2 cells were co-transduced with single-guide RNA/Cas9/green fluorescence protein (GFP)-expression vectors and single-stranded oligodeoxynucleotides using Amaxa Nucleofector 2b (Lonza, Cologne, Germany). Forty-eight hours thereafter, the cells were sorted as per GFP-positive expression using FACS Aria II (Becton Dickinson, Franklin Lakes, NJ), and the frequencies of mutation were estimated using the TIDER web tool^57^. Single-cell clones were subsequently isolated with the limiting dilution method. Each clone was expanded, and mutations were identified with direct sequencing of the target region.

### Erythroid differentiation of HUDEP-2 cells

HUDEP-2 cells differentiated into erythroid cells via co-culture with OP9 cells in Iscove’s Modified Dulbecco’s Medium (Sigma-Aldrich) supplemented with 20% FBS, insulin-transferrin-selenium (Thermo Fisher Scientific), 50 μg/mL L-ascorbic acid 2-phosphate (Sigma-Aldrich), 0.45 mM 1-thioglycerol (Sigma-Aldrich), 1% penicillin–streptomycin, 3 IU/mL erythropoietin, and 1 μg/mL doxycycline for 6 days. Optionally, 100 μM SFC (SBI Pharmaceuticals, Tokyo, Japan) or 1 mM ALA (SBI Pharmaceuticals) was added to the medium.

### Intracellular heme concentration

Intracellular heme concentration was quantified using a spectrofluorometer (RF-5300PC; SHIMADZU, Kyoto, Japan). Cells were harvested and centrifuged at 300*g* for 5 min. Cell pellets were suspended in 2 M oxalic acid (Sigma-Aldrich) and boiled at 100 °C for 30 min to dissociate protoporphyrin IX and iron from heme. Subsequently, fluorescence for protoporphyrin IX was measured with excitation at 400 nm and emission at 662 nm. Fluorescence for each unboiled sample was used as the endogenous control for protoporphyrin IX levels. Hemin (Sigma-Aldrich) was dissolved in 40% dimethyl sulfoxide (Wako Pure Chemical, Osaka, Japan) and was used as the standard solution.

### Quantitative RT-PCR analysis

Total RNA was purified using TRIzol Reagent (Thermo Fisher Scientific), followed by complementary DNA synthesis using ReverTra Ace qPCR RT Master Mix with gDNA Remover (TOYOBO, Osaka, Japan). Relative messenger RNA levels were quantified using quantitative RT-PCR with Quantitect SYBR Green PCR Master Mix (Qiagen, Hilden, Germany) and appropriate primers that are listed in Supplementary Table S2. The data were finally normalized relative to the *GAPDH* expression levels. Standard plasmids were constructed by cloning complementary DNA PCR amplicon of each gene into the pGEM-T Easy Vector (Promega, Madison, WI). The copy number for each standard plasmid was calculated as follows: copy number (copy/μL) = 6.02 × 10^23^ × [plasmid concentration (μg/μL)] × 10^−6^/[total plasmid size (base pair)] × 660.

### Cytospin

Cells were centrifuged at 500 rpm for 3 min using the Shandon Cytospin 4 Cytocentrifuge (Thermo Fisher Scientific). The cells were subsequently stained with May-Grunwald Giemsa stain (Merck KGaA, Darmstadt, Germany) and Prussian blue stain (Sigma-Aldrich). Ring sideroblasts were defined as erythroblasts wherein 5 or more siderotic granules cover at least one-third of the nucleus circumference, as per the recommendation by the International Working Group on Morphology of Myelodysplastic Syndrome^58^.

### Electron microscopy

Electron microscopy was performed as previously described^22^. Cells were fixed with 2% paraformaldehyde and 2.5% glutaraldehyde in 0.1 M cacodylate buffer. The cells were then post-fixed in 1% osmium tetroxide for 30 min at 4 °C, rinsed in 0.1 M cacodylate buffer containing 8% sucrose, dehydrated in a graded series of alcohol and propylene oxide, and finally embedded in epoxy resin. Ultrathin (75 nm) sections were prepared using an ultramicrotome (UC-7; Leica, Heerbrugg, Switzerland) and stained with uranyl acetate and lead citrate before viewing under an electron microscope (H-7600; Hitachi, Tokyo, Japan and JEM1400; Japan Electron Optics Laboratory, Tokyo, Japan).

### Expression profiling

Human Oligo chip 25 k (Toray, Tokyo, Japan) was used for expression profiling. Enrichment analyses were conducted using Metascape^59^.

### Flow cytometry

The cells were washed and resuspended in phosphate-buffered saline containing 3% FBS, followed by incubation with fluorescent-conjugated antibodies specific for CD49d (clone: 9F10), CD71 (clone: GA-R2), and CD235a (clone: M-A712) (Becton Dickinson). Propidium iodide (PI; Thermo Fisher Scientific) was used for the marking of dead cells. Liperfluo (Dojindo, Kumamoto, Japan) was used for the detection of cellular lipid peroxide. FerroFarRed (Goryo Chemical, Sapporo, Japan) was used for the detection of intracellular labile iron. Data were acquired with FACS Aria II or FACS Canto II (Becton Dickinson) and were analyzed using FlowJo version 7.6.5 software (TreeStar, Ashland, OR, www. flowjo. com).

### Luminol-enhanced chemiluminescence

Luminol-enhanced chemiluminescence that represents lipid peroxidation was measured using a chemiluminescence analyzer (CLA-FS4; Tohoku Electronic Industrial, Sendai, Japan) as previously described^60^.

### Western blotting

Ten million cells per 1 mm sodium dodecyl sulfate sample buffer (25 mM Tris, pH 6.8, 2% b-mercaptoethanol, 3% sodium dodecyl sulfate, 0.1% bromophenol blue, and 5% glycerol) were boiled at 100 °C for 10 min to prepare whole-cell extracts. Extracts from 1 × 10^5^ cells were loaded into sodium dodecyl sulfate polyacrylamide gel, and electrophoresis was performed followed by transfer to a Hybond-P polyvinylidene fluoride blotting membranes (GE Healthcare, Cleveland, OH). The membranes were blocked in tris-buffered saline with Tween 20 (Sigma-Aldrich) containing 5% milk for 1 h and subsequently incubated overnight with primary antibodies at the following concentrations: α-tubulin (CP06; EMD-Millipore, Billerica, MA) 1:2000, BACH1 (previously described^61^) 1:500, GCLM (ab126704; Abcam plc, Cambridge, UK) 1:1000, GCLC (ab53179; Abcam plc) 1:1000, ALAS2 (ab184964; Abcam plc) 1:1000 and ferritin (ab75973; Abcam plc) 1:1000. On the following day, the membranes were washed and incubated with 1:5000 goat anti-rabbit horseradish peroxidase-conjugated IgG (ab6721; Abcam plc) or goat anti-mouse horseradish peroxidase-conjugated IgG (ab6789; Abcam plc) for 1 h. The images were finally captured using ECL-Plus Kit (GE Healthcare) and CL-X Posure film (Thermo Fisher Scientific).

### ChIP-Seq analysis

We used ChIP-seq data from the GEO data set: BACH1 in K562, GSM935576; MAFK in K562, GSM935311; NFE2 in K562, GSM935414; and NFE2 in human erythroblasts, GSM1427076. The peak profiles were obtained from the ChIp-Atlas database (https://chip-atlas.org) and visualized using the Integrative Genomics Viewer (IGV version 2.8.2; Broad Institute, Cambridge, MA).

### Intracellular glutathione concentration

Thirty million differentiated HUDEP-2 cells were harvested and sonicated in 1 mL of cold buffer [0.2 M 2-(N-morpholino)ethanesulphonic acid, 50 mM phosphate, and 1 mM ethylenediaminetetraacetic acid]. After centrifugation at 10,000×*g* for 15 min at 4 °C, 800 μL of the supernatant was mixed with 800 μL of 10% (wt/vol) metaphosphoric acid (Sigma-Aldrich) solution. Proteins were precipitated via centrifugation at 2000×*g* for 2 min, and 1 mL of the supernatant was collected. Fifty microliters of 4 M triethanolamine solution (Sigma-Aldrich) was added and vortexed; subsequently, the total intracellular glutathione concentration was measured using the Glutathione Assay Kit (Cayman Chemical, Ann Arbor, MI). One hundred and fifty microliters of assay cocktail containing 5, 5′-dithio-bis-(2-nitrobenzoic acid), Ellman’s reagent, was added to 50 μL of each sample and incubated at room temperature in the dark. Twenty-five minutes thereafter, the absorbance was measured at 405 nm. Finally, the glutathione concentration was determined in reference to the total glutathione standard curve.

### Statistical analyses

GraphPad Prism 9 (GraphPad Software, San Diego, CA, www.graphpad.com) was used for all the statistical analyses.

## Supplementary Information

Supplementary Information 1.

Supplementary Information 2.

## Data Availability

All data generated or analyzed during this study are included in Supplementary Information files.
