# Supplementary material for: Congenital sideroblastic anemia model due to ALAS2 mutation is susceptible to ferroptosis
Source: Sci Rep. 2022 May 30;12:9024. doi: 10.1038/s41598-022-12940-9 (PMC9151922; doi:10.1038/s41598-022-12940-9)
Supplement: Supplementary file 2 — Supplementary Information 2. [file 41598_2022_12940_MOESM2_ESM.pdf]

Supplementary information for:

Congenital sideroblastic anemia model due to *ALAS2* mutation is susceptible to ferroptosis

Koya Ono[1], Tohru Fujiwara[1,2], Kei Saito[1], Hironari Nishizawa[3], Noriyuki Takahashi[2], Chie Suzuki[2], Tetsuro Ochi[1], Hiroki Kato[1], Yusho Ishii[4], Koichi Onodera[1], Satoshi Ichikawa[1], Noriko Fukuhara[1], Yasushi Onishi[1], Hisayuki Yokoyama[1], Rie Yamada[5], Yukio Nakamura[6], Kazuhiko Igarashi[3], Hideo Harigae[1,2,*]

[1] Department of Hematology, Tohoku University Graduate School of Medicine, Sendai, Japan
[2] Laboratory Diagnostics, Tohoku University Hospital, Sendai, Japan
[3] Department of Biochemistry, Tohoku University Graduate School of Medicine, Sendai, Japan
[4] Department of Rheumatology, Tohoku University Graduate School of Medicine, Sendai, Japan
[5] Tohoku Electronic Industrial Co., Ltd., Sendai, Japan
[6] Cell Engineering Division, RIKEN BioResource Research Center, Tsukuba, Ibaraki, Japan

* Corresponding. harigae@med.tohoku.ac.jp

Supplementary Figure S1

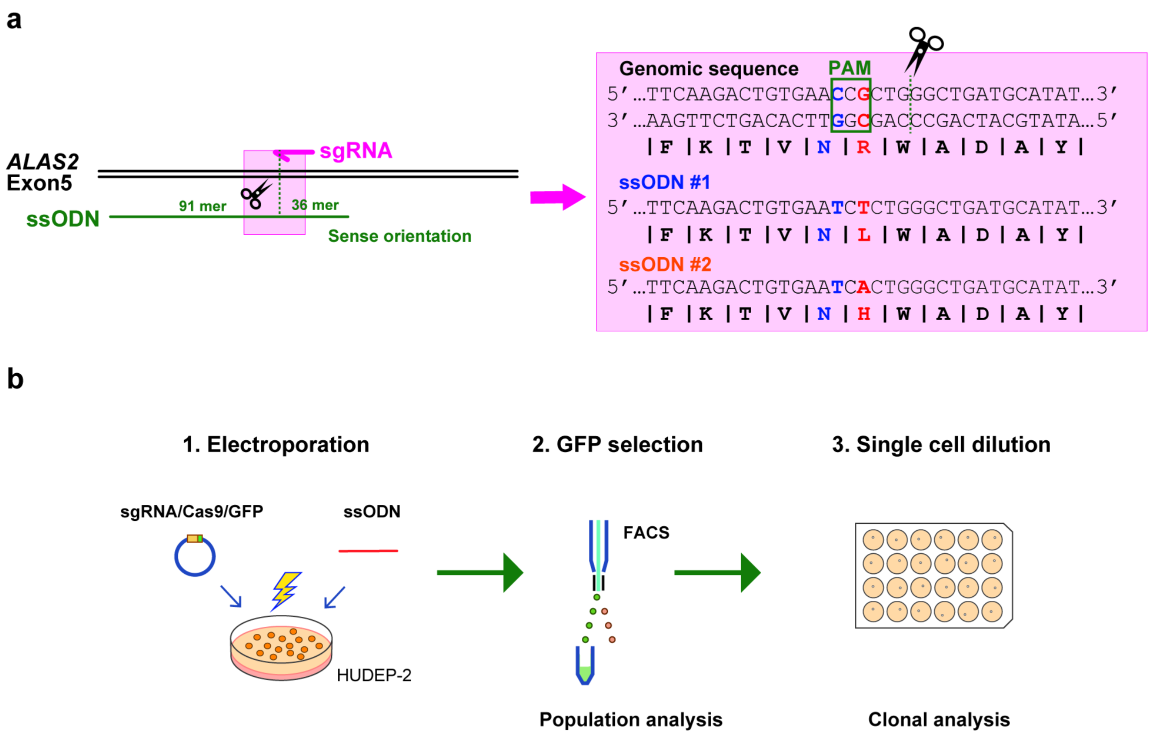

Figure S1. CRISPR/Cas9-based introduction of *ALAS2* R170L and R170H mutations. **(a)** Single-guide RNA (sgRNA: 5′- AGGGATATGCATCAGCCCAG -3′) was designed as a 20-nucleotide sequence complementary to the genomic region located upstream of protospacer adjacent motif (PAM) near arginine at amino acid residue 170 in *ALAS2*. The sgRNA was cloned into pGuide-it-ZsGreen1 Vector (Takara Bio, Shiga, Japan) that expresses Cas9 and green fluorescent protein (GFP). A homologous recombination templates were designed as 127-mer single-stranded oligodeoxynucleotides (ssODN) asymmetrically distributed from the Cas9-mediated cleavage site. In ssODN, single-nucleotide substitutions were induced to generate *ALAS2* R170L and R170H mutations. Silent mutations were also included to avoid continuous cleavage after successful mutation. **(b)** Experimental procedure for CRISPR/Cas9. FACS, fluorescence-activated cell sorter.

Supplementary Figure S2

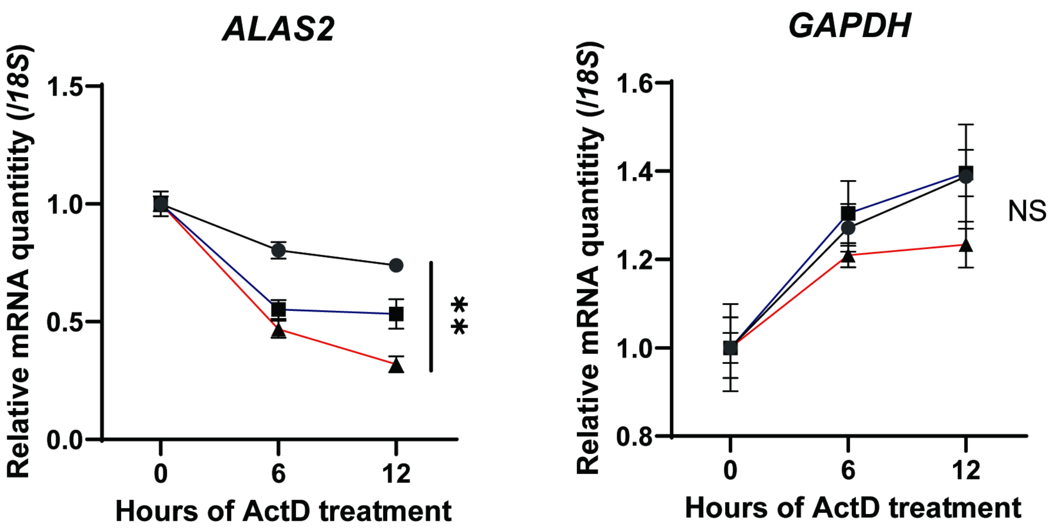

Figure S2. ***ALAS2*** **R170L and R170H mutations cause messenger RNA (mRNA) instability.** HUDEP^{WT}, HUDEP^{R170L}, and HUDEP^{R170H} after 6 day differentiation with sodium ferrous citrate were treated with 5 mg/mL of the transcription inhibitor actinomycin D (ActD; Nacalai Tesque, Kyoto, Japan). *ALAS2* mRNA levels were measured after 6 or 12 h of ActD treatment. *GAPDH* mRNA levels are shown as controls. The values are normalized to 18S ribosomal RNA and relative to time = 0. The averages ± standard error of the mean (n = 3) are shown. Each *P*-value was calculated using Tukey's test after a two-way analysis of variance. ***P* < 0.01; NS, not significant.

Supplementary Figure S3

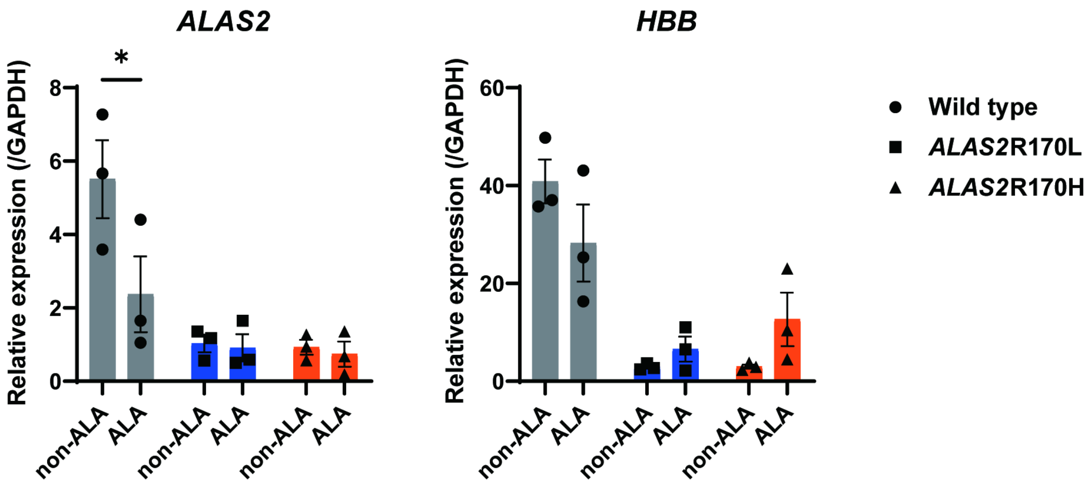

Figure S3. Effects of 5-aminolevulinic acid (ALA) supplementation on gene expression during erythroid differentiation of XLSA clones. Quantitative reverse transcriptase-polymerase chain reaction analysis for *ALAS2* and *HBB*, expression relative to *GAPDH* in $HUDEP^{WT}$, $HUDEP^{R170L}$, and $HUDEP^{R170H}$ after 6 day differentiation with or without ALA supplementation (n = 3). The graphs were plotted using GraphPad Prism 9 (GraphPad Software, San Diego, CA, www.graphpad.com). The error bars represent the standard error of the mean. Each *P*-value was calculated using Šidák's test after a two-way analysis of variance. $^*P < 0.05$.

Supplementary Figure S4

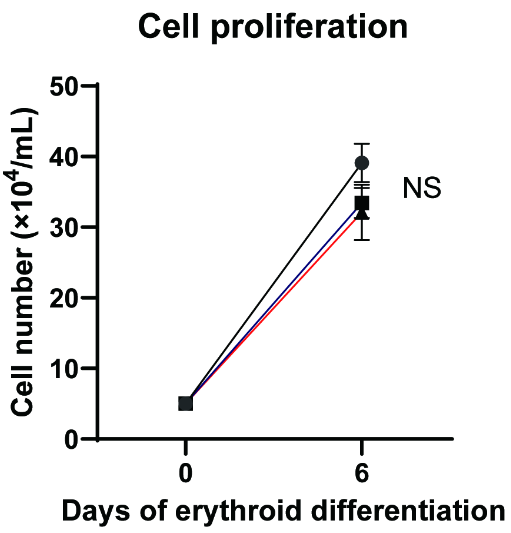

Figure S4. XLSA clones proliferate normally during erythroid differentiation. Cell numbers of $HUDEP^{WT}$, $HUDEP^{R170L}$, and $HUDEP^{R170H}$ were counted before and after 6 day differentiation with sodium ferrous citrate. Each *P*-value was calculated using Tukey's test after a two-way analysis of variance. NS, not significant.

Supplementary Figure S5

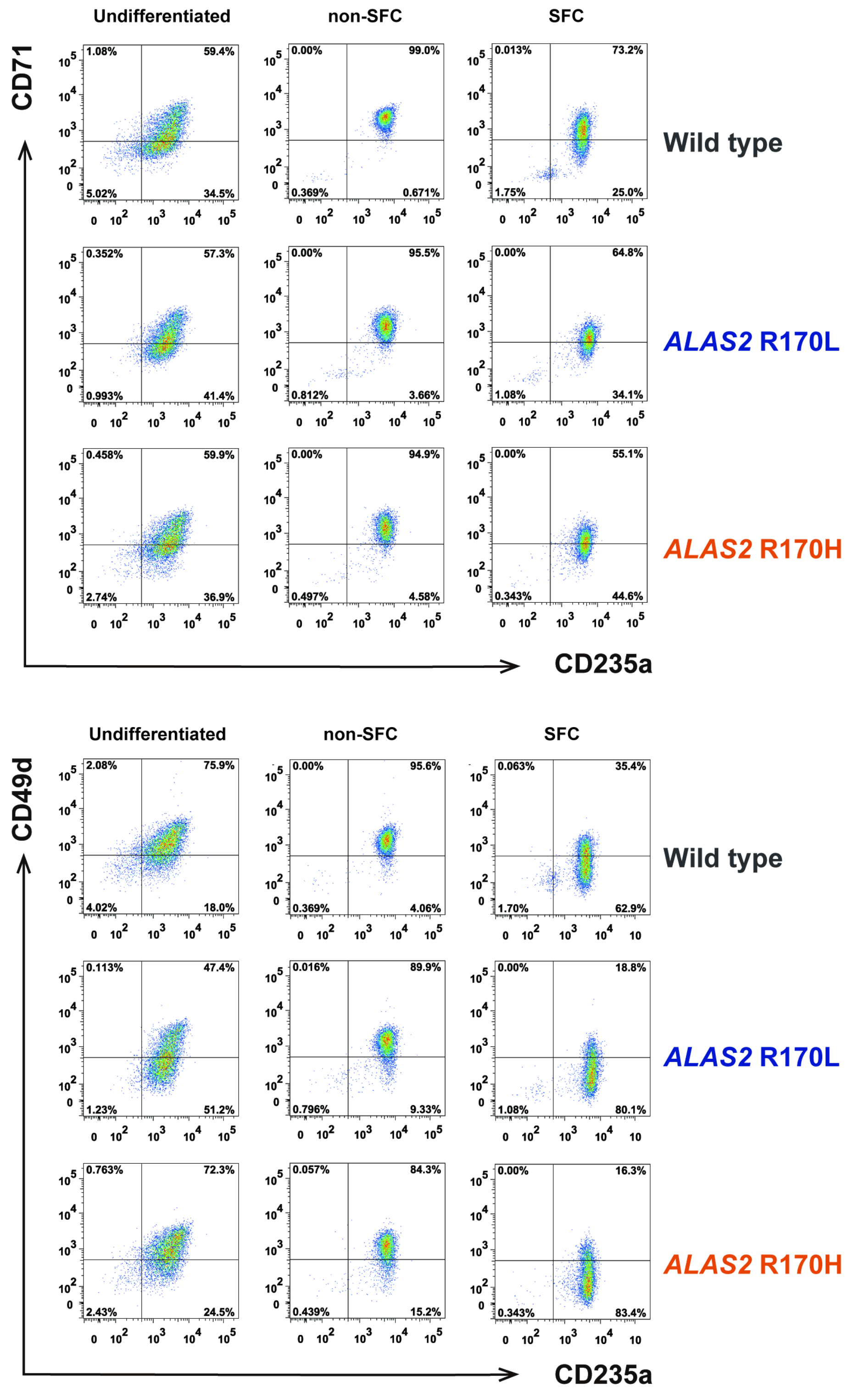

Figure S5. Flow cytometric analysis of HUDEP^{WT}, HUDEP^{R170L}, and HUDEP^{R170H} stained with anti-CD71, anti-CD235a, and anti-CD49d antibodies. The representative

data of three independent experiments, analyzed using FlowJo version 7.6.5 software (TreeStar, Ashland, OR, www. flowjo. com), are shown. SFC, sodium ferrous citrate.

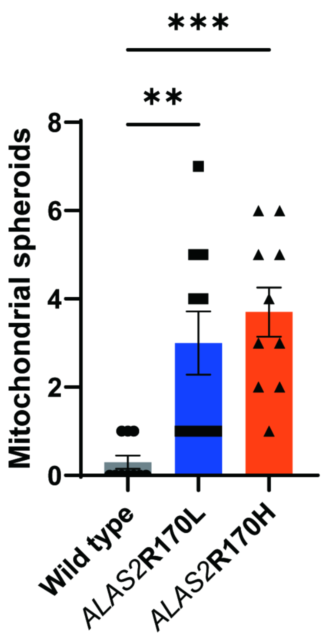

Figure S6. Count of mitochondrial spheroids of HUDEP-2 clones after 6 day differentiation with sodium ferrous citrate. For quantification, ten cells per condition were counted in randomly selected fields. The graphs were plotted using GraphPad Prism 9 (GraphPad Software, San Diego, CA, www.graphpad.com). The error bars represent the standard error of the mean. Each *P*-value was calculated using Tukey's test after a two-way analysis of variance. **$P < 0.01$, ***$P < 0.001$.

Supplementary Figure S7

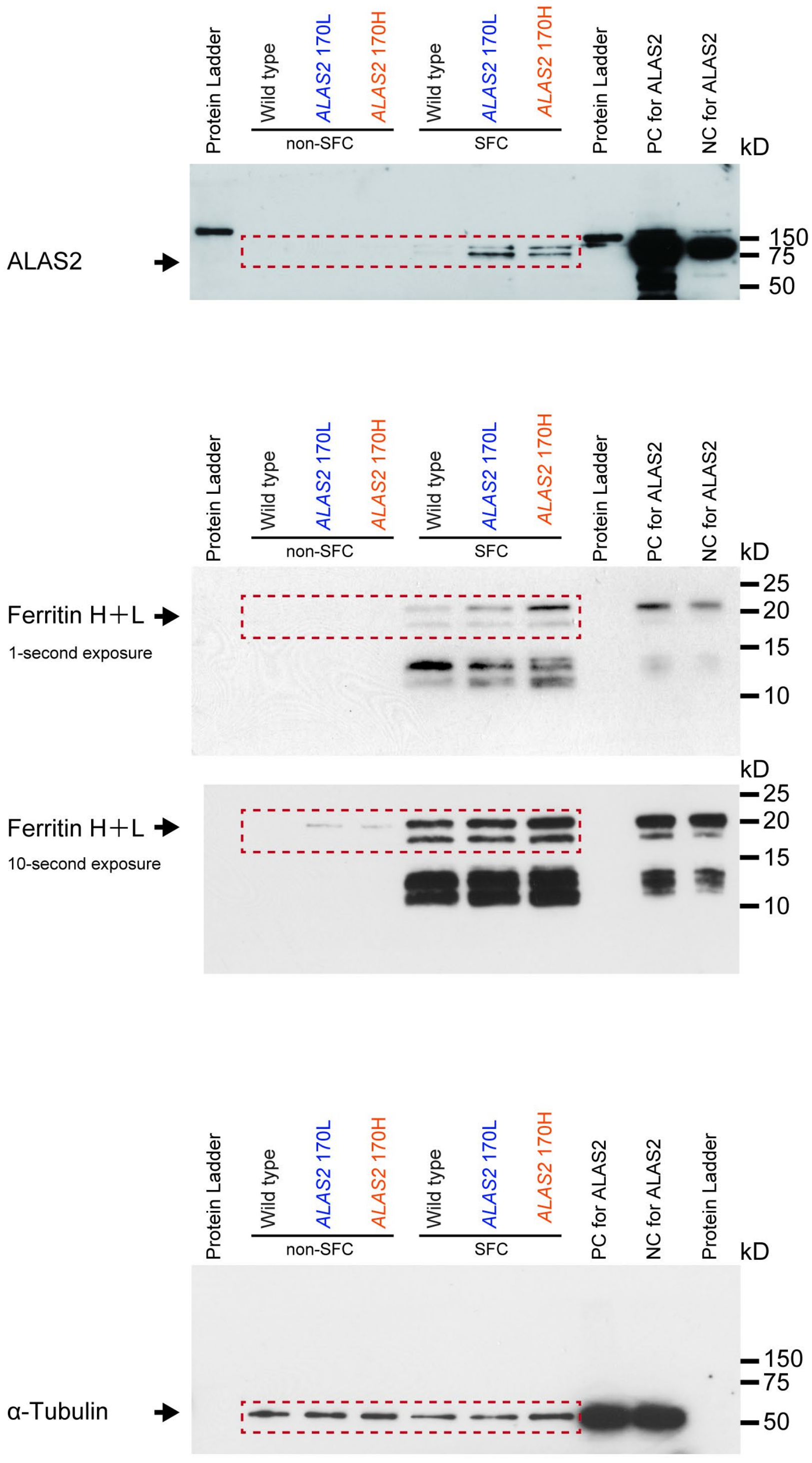

Figure S7. Original Western blots of Figure 3c. Precision Plus Protein WesternC Blotting Standards (Bio-Rad, Hercules, CA) was used as Protein Ladder. For the final figures, Adobe

Photoshop was used to crop the indicated bands (red dashed lines) and the bands were placed in Figure 3c. NC, negative control; PC, positive control.

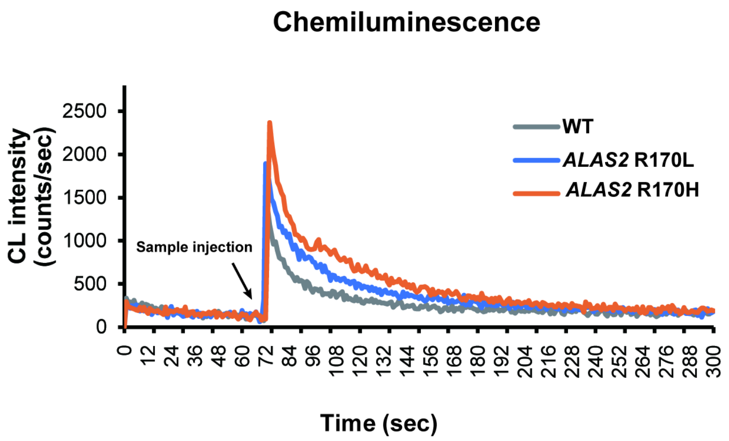

Figure S8. XLSA clones show increased lipid peroxidation. Luminol-enhanced chemiluminescence (CL) that represents lipid peroxidation was measured using a CL analyzer (CLA-FS4; Tohoku Electronic Industrial, Sendai, Japan) as previously described[1]. Time course of luminol-enhanced CL of samples that are added to the CL reagent containing 2.0 mg/L luminol and 10 mg/L cytochrome c in 50 mM borate buffer (pH 10.0). Lysates of HUDEP^{WT}, HUDEP^{R170L}, and HUDEP^{R170H} after 6 day differentiation with sodium ferrous citrate were used as samples.

Supplementary Figure S9

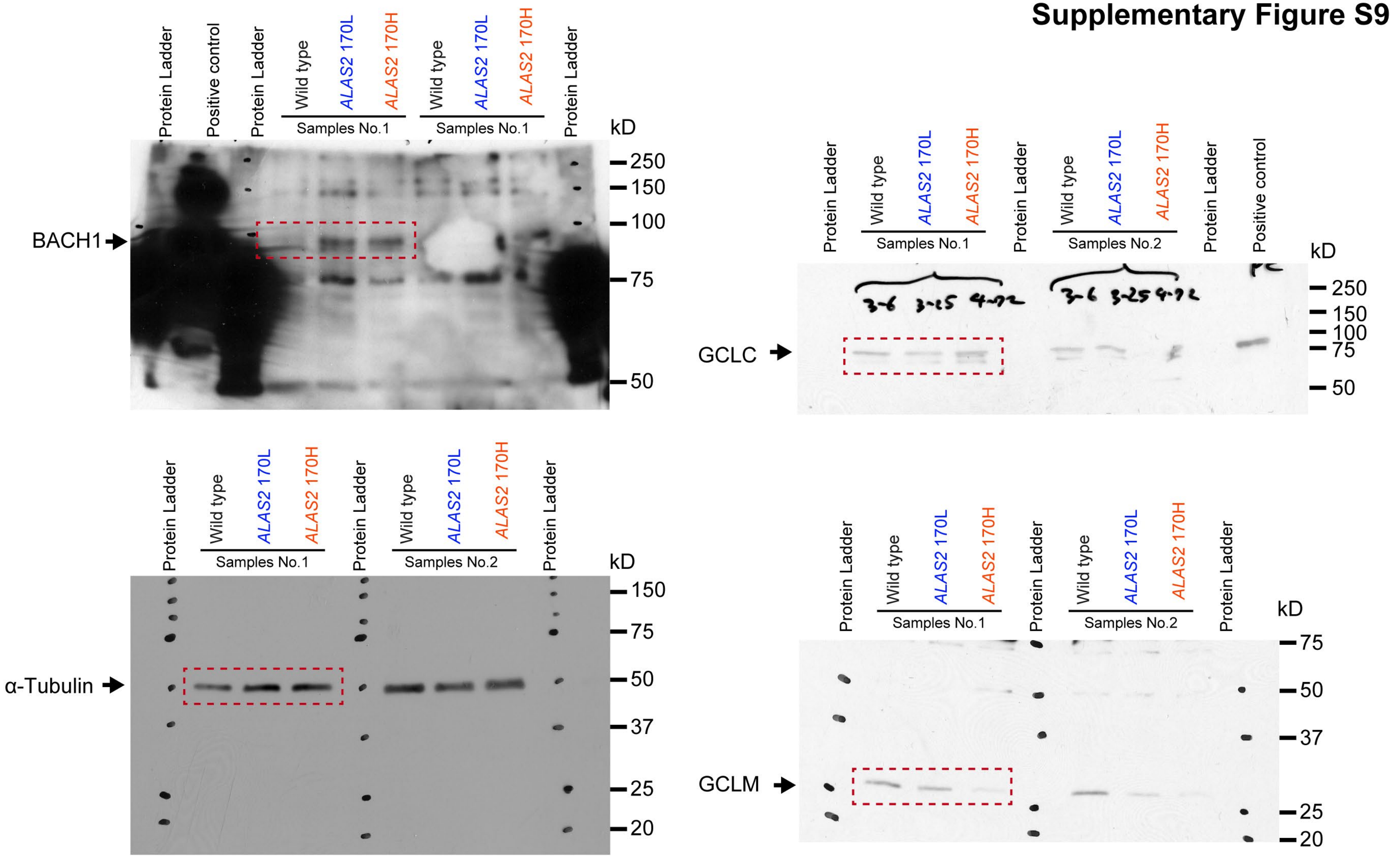

Figure S9. Original Western blots of Figure 6b. Precision Plus Protein WesternC Blotting Standards (Bio-Rad, Hercules, CA) was used as Protein Ladder. For the final figures, Adobe Photoshop was used to crop the indicated bands (red dashed lines) and the bands were placed in

Figure 6b.

Supplementary Figure S10

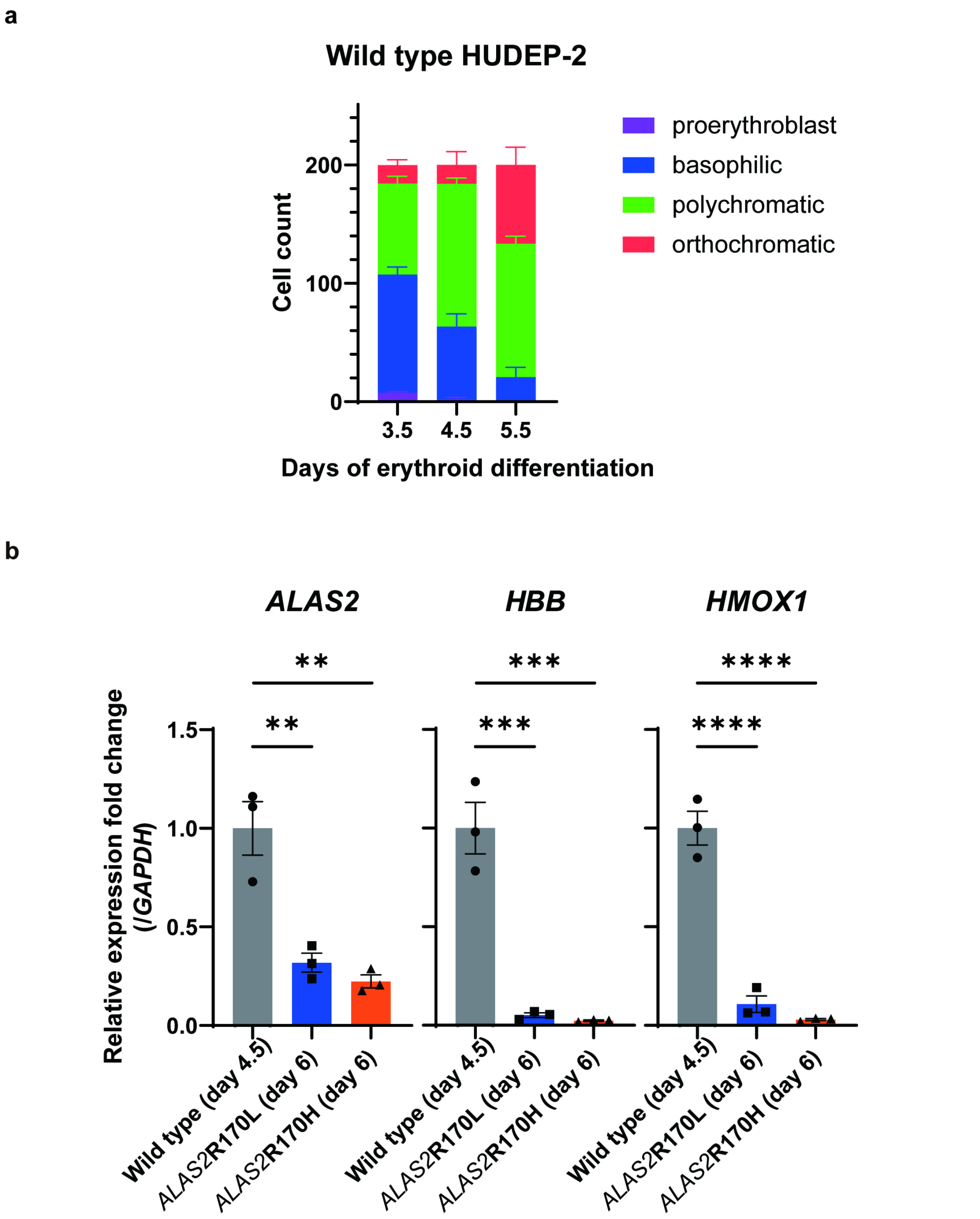

Figure S10. Gene expression levels in XLSA clones are much lower than those in less-differentiated HUDEP^{WT}. (a) Count of erythroid progenitor cells derived from HUDEP-2 clones after 3.5, 4.5, and 5.5 day differentiation (n = 3). May-Giemsa staining was used for quantification. The graphs were plotted using GraphPad Prism 9 (GraphPad Software, San

Diego, CA, www.graphpad.com). **(b)** Quantitative reverse transcriptase-polymerase chain reaction analysis for *ALAS2*, *HBB* and *HMOX1* in HUDEP-2 clones after differentiation with sodium ferrous citrate. Fold changes in messenger RNA levels which are normalized relative to the *GAPDH* levels are shown. The error bars represent the standard error of the mean. Each *P*-value was calculated using Tukey's test after a one-way analysis of variance. $^{**}P < 0.01$, $^{***}P < 0.001$, $^{****}P < 0.0001$.

Supplementary Figure S11

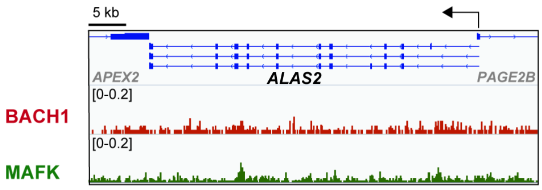

Figure S11. Chromatin immunoprecipitation with sequencing (ChIP-seq) analysis of the binding of BACH1 and MAFK for the gene region in K562 for *ALAS2*. We used ChIP-seq data from GEO (Gene Expression Omnibus) data set: BACH1 in K562, GSM935576; and MAFK in K562, GSM935311.

Summary of potential off-target sites in *ALAS2* R170

Target sequence: AGGGATATGCATCAGCCCAG

Locus	Sequence*	Chromosome Position	Score	PCR Primers
Off-target #1	AGGG**C**T**C**T**T**CATCAGCCCAGTGG -- hit<br>AGGGATATGCATCAGCCCAGNGG -- query	Chr10:62374724-62374746	0.77	FWD: GCAGCAACAAGTCGGGTAAGAG<br>REV: AGGTGCAAATGGTCCCCAGG
Off-target #2	AG**G**GGATGCATCAGCCCAGAGG -- hit<br>AGGGTATGCATCAGCCCAGNGG -- query	Chr11:11519165-11519186	0.91	FWD: TCCTCTCCTGGCTGTGGCTA<br>REV: GGACTGCATCAAACAGGCTTCCA
Off-target #3	A**A**GGA**A**ATGC**T**TCAGCCCAGAGG -- hit<br>AGGGATATGCATCAGCCCAGNGG -- query	Chr18:32452555-32452577	1.04	FWD: TAAAACCCCCTTCTCTGGATCCTC<br>REV: GGACCCCAGTGAATTCTGTTGC
Off-target #4	AG**A**ATA**G**GCATCAGCCCAGAGG -- hit<br>AGGATATGCATCAGCCCAGNGG -- query	Chr7:68874509-68874530	1.08	FWD: CAGAGGCATCCTTGGGAGAG<br>REV: GCTGGGATGACTCCTGATACAATG
Off-target #5	AGGA**CT**TGCATCAGCCCAGTGG -- hit<br>AGGATATGCATCAGCCCAGNGG -- query	Chr9:112938137-112938158	1.08	FWD: GTGTGCAACAGTAGCCTCTGCAT<br>REV: CCTCATCTCTAGAGGCTTTGTGAC

* *Red letters* indicate mismatched bases

Table S1. Summary of off-target analysis in *ALAS2* R170. Potential off-target sites were identified using the COSMID web tool[2,3], and top five candidate sites were listed for analysis. "Score" in this table shows the level of risk for off-target mutations. The possibility of an off-target effect for each clone was excluded via direct sequencing around the candidate sites using indicated polymerase chain reaction (PCR) primers.

Supplementary Table S2

Primers for quantitative RT-PCR

Primers	Direction	Sequences (5'→3')
ALAS2	Forward	CCTCAGATGATGGAAGATTTTG
	Reverse	TCATGAGCTCAAAGTGTACAGGA
HBB	Forward	TCCTGAGGAGAAGTCTGCCGT
	Reverse	GGAGTGGACAGATCCCCAAAG
HBA	Forward	ACCATGGTGCTGTCTCCTG
	Reverse	GAAGTGCGGGAAGTAGGTCTT
TfR1	Forward	AAACTGGACAGCACAGACTTCA
	Reverse	CGCAAGATTTTCATCTTTTTGA
DMT1	Forward	GCTGAAGTATGTCACCGTCAGT
	Reverse	ATGACTTCTTGCATGTCTGAGC
HMOX1	Forward	ATGAACTCCCTGGAGATGACTC
	Reverse	CCTTGGTGTCATGGGTCAG
GCLM	Forward	CAACTAGAAGTGCAGTTGACATGG
	Reverse	TCACAGAATCCAGCTGTGCAA
GCLC	Forward	TCCAGGTGACATTCCAAGCC
	Reverse	GAAATCACTCCCCAGCGACA
SLC40A1	Forward	CCTGTTAACAAGCACCTCAGC
	Reverse	TTGCAGAGGTCAGGTAGTCG
FTH1	Forward	AACTACCACCAGGACTCAGAGG
	Reverse	ATTTGGCAAAGTTCTTCAAAGC
FTL	Forward	GCCTCCTACACCTACCTCTCTC
	Reverse	CTGGTTTTGCATCTTCAGGAG
GAPDH	Forward	GAAGGTCGGAGTCAACGGATTT
	Reverse	GAATTTGCCATGGGTGGAAT
18S	Forward	CGCCGCTAGAGGTGAAATTCT
	Reverse	CGAACCTCCGACTTTCGTTCT

Table S2. Sequences of primers used in this study. RT-PCR, reverse transcriptase-polymerase chain reaction.

References

1 Kohno, M. *et al.* Early diagnosis of cancer by detecting the chemiluminescence of hematoporphyrins in peripheral blood lymphocytes. *Tohoku J Exp Med* **216**, 47-52, doi:10.1620/tjem.216.47 (2008).

2 Cradick, T. J., Qiu, P., Lee, C. M., Fine, E. J. & Bao, G. COSMID: A Web-based Tool for Identifying and Validating CRISPR/Cas Off-target Sites. *Mol Ther Nucleic Acids* **3**, e214, doi:10.1038/mtna.2014.64 (2014).

3 Sakuma, T., Sakamoto, T. & Yamamoto, T. All-in-One CRISPR-Cas9/FokI-dCas9 Vector-Mediated Multiplex Genome Engineering in Cultured Cells. *Methods in molecular biology (Clifton, N.J.)* **1498**, 41-56, doi:10.1007/978-1-4939-6472-7_4 (2017).
